# Biphasic effects of autophagy on decompression bubble‐induced endothelial injury

**DOI:** 10.1111/jcmm.14672

**Published:** 2019-09-12

**Authors:** Mengmeng Wang, Kun Zhang, Shaojie Nie, Guoyang Huang, Hongjie Yi, Chunyang He, Peter Buzzacott, Weigang Xu

**Affiliations:** ^1^ Department of Diving and Hyperbaric Medicine Naval Medical University Shanghai China; ^2^ Administration Office for Undergraduates Naval Medical University Shanghai China; ^3^ Department of Hyperbaric Oxygen Changhai Hospital Shanghai China; ^4^ Department of Hyperbaric Oxygen General Hospital in Western Theater of Operations Chengdu China; ^5^ School of Sports Science, Exercise and Health The University of Western Australia Crawley Perth Australia

**Keywords:** autophagy, bubble, decompression sickness, endothelial cell

## Abstract

Endothelial dysfunction induced by bubbles plays an important role in decompression sickness (DCS), but the mechanism of which has not been clear. The present study was to investigate the role of autophagy in bubble‐induced endothelial injury. Human umbilical vein endothelial cells (HUVECs) were treated with bubbles, autophagy markers and endothelial injury indices were determined, and relationship strengths were quantified. Effects of autophagy inhibitor 3‐methyladenine (3‐MA) were observed. Bubble contact for 1, 5, 10, 20 or 30 minutes induced significant autophagy with increases in LC3‐II/I ratio and Beclin‐1, and a decrease in P62, which correlated with bubble contact duration. Apoptosis rate, cytochrome C and cleaved caspase‐3 increased, and cell viability decreased following bubble contact for 10, 20 or 30 minutes, but not for 1 or 5 minutes. Injuries in HUVECs were correlated with LC3‐II/I ratio and partially reversed by 3‐MA in 10, 20 or 30 minutes contact, but worsened in 1 or 5 minutes. Bubble pre‐conditioning for 1 minutes resulted in increased cell viability and decreased apoptosis rate compared with no pre‐conditioning, and 30‐minutes pre‐conditioning induced opposing changes, all of which were inhibited by 3‐MA. In conclusion, autophagy was involved and played a biphasic role in bubble‐induced endothelial injury.

## 1 INTRODUCTION

Decompression sickness (DCS) is a medical concern that threatens the safety of divers and is associated with bubbles that may appear during or after decompression, even after strictly following diving protocols.^1^, ^2^ Endothelial dysfunction occurs following decompression in divers,^3^, ^4^ and endothelial protective agents have proven beneficial to DCS in animal models.^5^, ^6^, ^7^ It is now widely accepted that endothelial injury triggered by bubbles plays an important role in the pathophysiology of DCS.^8^, ^9^, ^10^ However, the precise mechanisms of endothelial dysfunction have not been fully understood.

Autophagy, a cellular lysosome‐dependent degradation process, widely participates in physiological and pathological activities of endothelial cells such as proliferation, migration and angiogenesis.^11^ The role of autophagy involved in endothelial injury in atherosclerosis, ischaemia‐reperfusion, hepatopulmonary syndrome and other diseases has been well studied.^12^, ^13^, ^14^ It can be induced by both physical and chemical stimuli, including shear stress, calcium and oxidative stress.^15^, ^16^, ^17^ Intravascular bubbles can disrupt blood flow or even occlude vessels, resulting in altered shear stress and ischaemia‐reperfusion injury.^18^, ^19^ Bubbles have also been reported to induce increased intracellular calcium in human umbilical vein endothelial cells (HUVECs).^20^, ^21^, ^22^ We have been suggested that bubbles could activate endothelial autophagy.

The purpose of this study was to explore the role of autophagy in bubble‐induced endothelial injury. Markers of autophagy and endothelial injury were determined following bubble treatment for different durations and the relationships between autophagy and endothelial injury were explored.

## 2 MATERIALS AND METHODS

### 2.1 Cell culture and treatments

Human umbilical vein endothelial cells were purchased from American Type Culture Collection (ATCC) (Cat. No. CRL‐1730). Cells were cultured in high glucose Dulbecco's modified Eagle medium (DMEM; Gibco; Cat. No. 11965) containing 10% foetal bovine serum (FBS; Hyclone; Cat. No. 0025) and incubated in a humidified atmosphere of 5% carbon dioxide (CO_2_). For all of the experiments, HUVECs in passage 3 ~ 7 were used. The experiments were carried out in two parts.

#### 2.1.1 Part 1: Effects of different durations of bubble contact on HUVECs autophagy and cell function

After exposure to bubbles for 30 minutes, autophagy markers LC3‐II/I ratio (the ratio of LC3‐II to LC3‐I), Beclin‐1 and P62 were determined at 2, 4, 8, 12 and 24 hours by Western blotting (WB). Since the expressions of these proteins were consistent after different intensities of stimuli, the peak or valley time were selected as the follow‐up detection time.^23^, ^24^ The number of autophagosomes was determined by transmission electron microscopy (TEM). LC3‐II/I ratio, Beclin‐1 and P62 were further detected following bubble contact for different durations. Cell viability, apoptosis rate and expressions of cytochrome C and cleaved caspase‐3 were determined prior to the experiment for assessing endothelial function at 12 hours following bubble contact. The relationships between autophagy markers and cell damage were quantified, and the effects of autophagy inhibitor 3‐methyladenine (3‐MA) on endothelial function following bubble contact were tested. 3‐MA was purchased from Sigma‐Aldrich (St. Louis, MO, USA, Cat. No. S2767) and was directly dissolved into the culture medium at the concentrations of 5 mmol/L for 24 hours to inhibit autophagy.^25^

#### 2.1.2 Part 2: Effects of a pre‐conditioning short contact on a second long damaging contact by bubbles in HUVECs

In *Part 1,* bubble contact for 1 and 5 minutes showed no effect on the observed parameters; however, the pre‐treatment of 3‐MA induced detectable endothelial damage. We hypothesized that a previous mild bubble contact would increase, while long‐time contact would decrease, the ability of HUVECs to counteract a second damaging contact. HUVECs were exposed to bubbles for 30 minutes following a bubble contact pre‐treatment for 1 or 30 minutes with an 8‐hours interval. Cell viability and apoptosis rate were determined. The effects of 3‐MA pre‐treatment before the first bubble contact were also investigated to clarify the role of autophagy.

### 2.2 Bubble contacting

**Figure 1:**
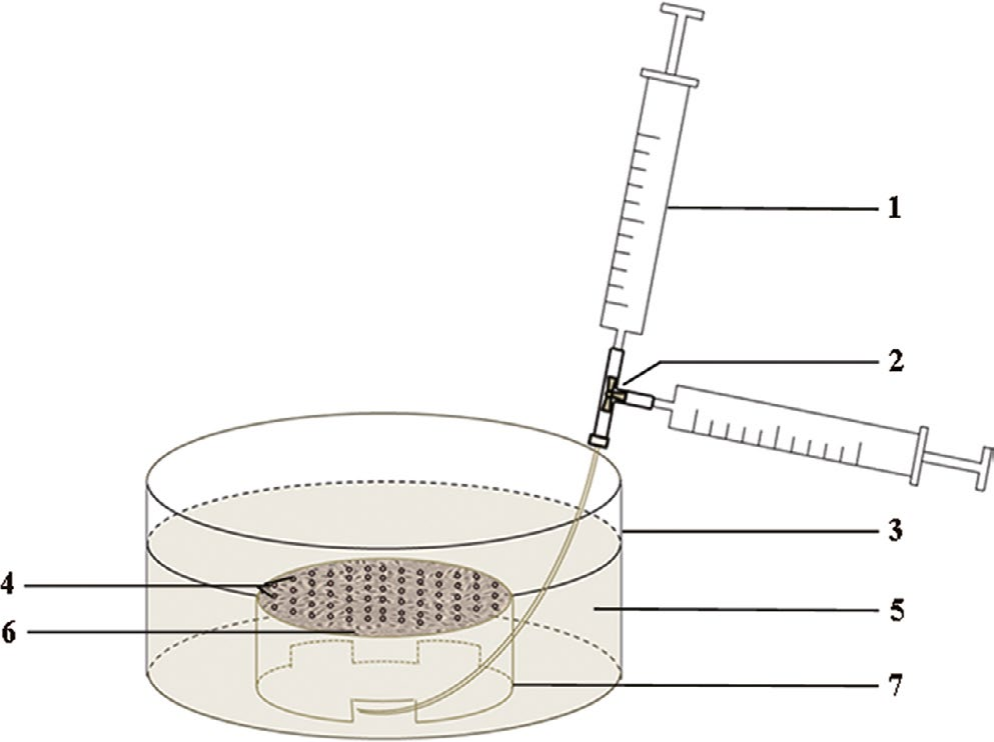
The schematic diagram of bubble contacting. 1. Syringe, 2. Medical tee tube, 3. Outer dish, 4. Bubbles, 5. DMEM medium, 6. HUVECs, 7. Inner dish

Bubble contact was performed either in 1.0 cm^2^ dishes or in 96‐well plates (Baisai Biotechnology). HUVECs (1 × 10^5^ cells/mL) were seeded in a dish. After cells grew to form a monolayer, the dish was turned over and immersed in the medium in a larger dish (the outer dish; Figure 1). When performed with 96‐well plates, 300‐μL medium was added into each well and the plates were turned over. As a result of surface tension, the medium in the wells did not flow out. Bubble medium was produced by alternatively depressing for 10× two 1‐mL syringes connected via a medical tee tube (Figure1). The two syringes contained 0.4‐mL DMEM medium and 0.2‐mL mixed gas (nitrogen with 16% CO_2_), respectively. Bubble medium was injected into the inner dish or wells to contact with the cell layer. The diameters of bubbles were detected using an DMi8 inverted microscope (Leica Microsystems). Four different areas were selected randomly from each dish or well, and bubbles were examined using a computer image analysis system (Smart Scape, Furi Science & Technology Co., Ltd). After treating for specific durations in the incubator, the inner dish or the plate was turned back and then bubble medium was removed.

### 2.3 Cell viability assay

Cell viability assay was performed in 96‐well plates using cell counting kit‐8 (CCK‐8; Dojindo Laboratories; Cat. No. GB707). Twelve hours following bubble contact, 10‐μL CCK‐8 regents were added into each well and incubated at 37°C in the dark for 2‐3 hours. The absorbance at 450 nm (A_450_) was measured using a Synergy HT auto‐microplate reader (Bio‐Tek). The percentage of cell viability was calculated as (A_450 treated_ − A_450 blank_/A_450 normal_ − A_450 blank_) × 100%. Cell viability of the untreated group was standardized as 100%.

### 2.4 Cell apoptosis assay

Cell apoptosis was determined on 35‐mm‐diameter dishes using Annexin‐V‐fluorescein isothiocyanate/propidium iodide (AV‐FITC/PI) apoptosis detection kit (Invitrogen; Cat. No. V13242). Twelve hours following bubble contact, cells were collected, washed twice with ice‐cold PBS and resuspended in 400‐µL binding buffer. The suspension was stained with 5‐µL AV‐FITC and 5‐µL PI dyes in the dark for 15 minutes. Apoptosis rate was determined by CytoFLEX flow cytometry (Beckman Coulter) and was calculated by the percentage of total cells that were early or late apoptotic cells (AV‐FITC+/PI − and AV‐FITC+/PI+). Apoptosis rate of the reference group was standardized as 100%.

### 2.5 Transmission electron microscopy observation

Transmission electron microscopy was used to directly observe the formation of autophagosome. According to the peak time of LC3‐II/I ratio and Beclin‐1, 8 hours following bubble exposure, cells were fixed in 2.5% glutaraldehyde at 4°C for 4 hours. Samples were then post‐fixed in 1% osmium tetroxide for 2 hours at 20°C, dehydrated through graded ethanol and then embedded in epoxy resin. Ultra‐thin sections (80 nm) were double stained using lead citrate and uranyl acetate and were observed under a Tecnai G^2^ transmission electron microscope (FEI, Hillsboro).

### 2.6 Western blotting analysis

Western blotting analysis was performed on 35‐mm‐diameter dishes to detect protein expression. Following bubble treatment, cells were washed twice with cold PBS; then, 100 μL cold RIPA buffer was added containing 10% protease inhibitor for 20 minutes, all reagents were purchased from Sigma, St Louis, MO, USA. The lysates were sonicated and then centrifuged at 14 000 × g for 10 minutes at 4˚C. The protein content was determined using a BCA detection kit (Beyotime). Equal amounts of protein (20 μg) were separated by 12% SDS‐PAGE gels (Beyotime) for 30 minutes at 80 V, followed by 50 minutes at 120 V and sequentially transferred to Polyvinyldifluoridine membrane (Millipore). After blocking with 5% no‐fat milk for 1 hours, the protein bands were incubated with the respective primary antibody against microtubule‐associated protein 1 light chain 3 (LC3; Cat. No. 3868; 1:1000 dilution), Beclin‐1 (Cat. No. 3738; 1:1000 dilution), cytochrome C (Cat. No. 4272; 1:1000 dilution), cleaved caspase‐3 (Cat. No. 9661; 1:1000 dilution) or β‐Actin (Cat. No. 4967; 1:1000 dilution) at 4°C overnight. The bands were washed three times and incubated with IRDye 800CW anti‐rabbit IgG (H&L) secondary antibody (Cat. No. 3686; 1:1000 dilution) for 1 hours at room temperature and then imaged using Licor Odyssey 9120 Imaging System with Warranty (Li‐Cor), the intensity of each band was analysed using Odyssey 3.0 analytical software (Li‐Cor). All antibodies were purchased from Cell Signaling Technology, Inc.

### 2.7 Statistical analysis

Data are expressed as mean ± SD (standard deviation). Differences between two groups were analysed by the independent sample *t* test or Mann‐Whitney *U* test. Differences among multiple groups were analysed by one‐way ANOVA followed by LSD*‐t* or Dunnett's T3 test. Linear correlations among autophagy markers and bubble contact durations or endothelial injury indices were assessed by Pearson correlation test. Statistical significance was defined at *P* < .05.

## 3 RESULTS

### 3.1 Effects of bubble contact on autophagy in HUVECs

**Figure 2:**
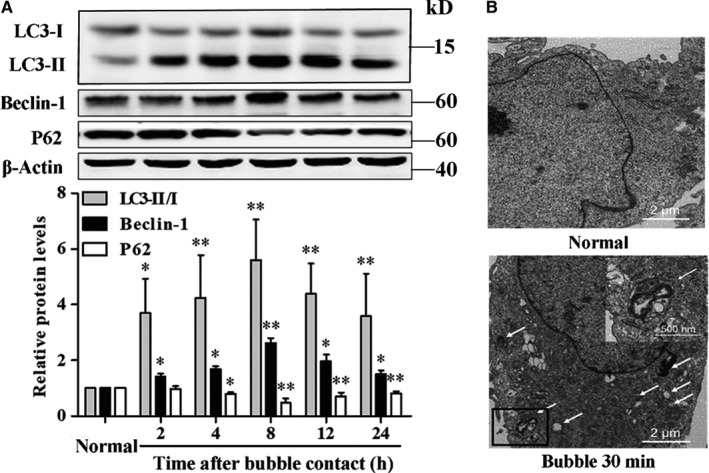
Autophagy in HUVECs was induced by bubble contact. A, LC3‐II/I ratio, Beclin‐1 and P62 were determined by WB at 2, 4, 8, 12 or 24 h after exposure to bubbles for 30 min. Values are expressed as mean ± SD, n = 4 for each group. ^*^ *P* < .05, ^**^ *P* < .01 vs Normal. B, Typical autophagosomes, indicated by white arrows, were detected by TEM at 8 h after bubble contact for 30 min

As shown in Figure 2A, after treating with bubbles for 30 minutes, LC3‐II/I ratio and Beclin‐1 gradually increased and peaked at 8 hours, while P62 decreased and reached the nadir at 8 hours too (*P* < .01). TEM analysis in Figure 2B showed that the number of autophagosomes in HUVECs significantly increased following bubble contact. Thus, 8 hours following bubble treatment was taken as the determination time‐point of autophagy in the later experiments.

**Figure 3:**
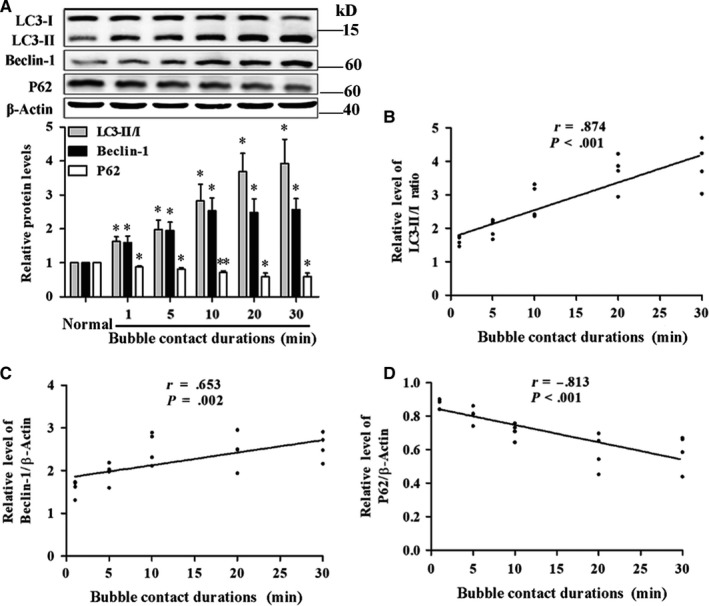
Relationships between autophagy and bubble contact duration. A, LC3‐II/I ratio, Beclin‐1 and P62 were detected by WB at 8 h after bubble contact for different durations. Values are expressed as mean ± SD, n = 4 for each group. ^*^ *P* < .05, ^**^ *P* < .01 vs Normal. B‐D, The correlations between bubble contact duration and LC3‐II/I ratio, Beclin‐1 and P62. Values are expressed as mean ± SD, n = 4 for each bubble contact duration and the total sample size was 20

The effects of different bubble contact durations on autophagy are shown in Figure 3A. Bubble contact for 1, 5, 10, 20 or 30 minutes induced significant increases in LC3‐II/I ratio and Beclin‐1 and a decrease in P62 (*P* < .05). Linear regression revealed that the peak value of LC3‐II/I ratio and Beclin‐1, and the valley value of P62 correlated with bubble contact durations (*P* < .01, Figure 3B‐D). Among them, LC3‐II/I ratio correlated best with bubble contact (*r* = 0.874) and was chosen to be the autophagy marker in the subsequent experiments.

### 3.2 The role of autophagy in bubble‐induced endothelial injury

**Figure 4:**
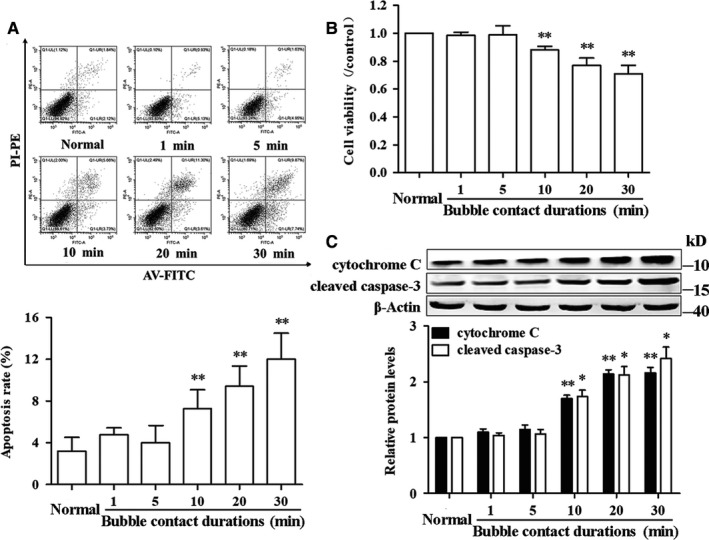
Bubble contact‐induced changes in endothelial injury markers. HUVECs were contacted with bubbles for 1, 5, 10, 20 or 30 min and endothelial markers were determined 12 h later. A, Apoptosis rate was determined using AV‐FITC/PI detection kit. B, Cell viability was determined using CCK‐8 assay kit. C, Cytochrome C and cleaved caspase‐3 were determined by WB. Values are expressed as mean ± SD, n = 4 for each group. ^*^ *P* < .05, ^**^ *P* < .01 vs Normal

**Figure 5:**
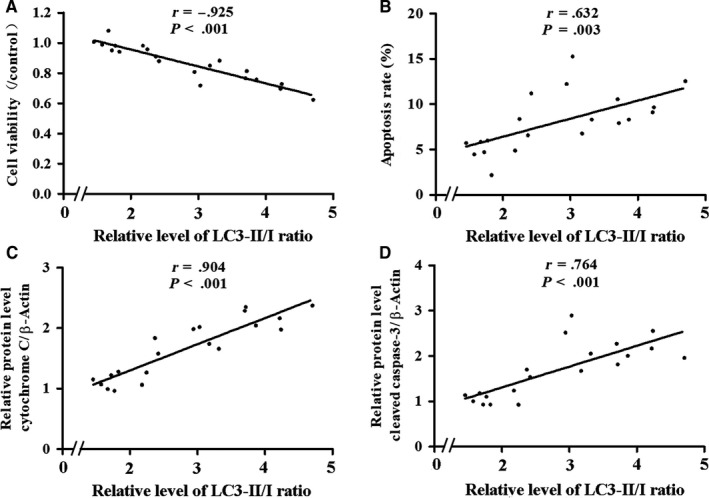
Correlation analysis between endothelial markers and LC3‐II/I ratio. Endothelial markers and LC3‐II/I ratio were determined following bubble treatment for 1, 5, 10, 20 or 30 min. Values are expressed as mean ± SD, n = 4 for each bubble contact duration and the total sample size was 20

Bubble contact for 10, 20 or 30 minutes induced a significant decrease in cell viability and remarkable increases in apoptosis rate, cytochrome C and cleaved caspase‐3 (Figure 4, *P* < .05). No difference was detected in the 1 and 5 minutes groups (*P* > .05). Further analysis showed a significant negative correlation between LC3‐II/I ratio and cell viability (*P* < .01), and apoptosis rate, cytochrome C and cleaved caspase‐3 correlated positively with LC3‐II/I ratio (Figure 5, *P* < .01).

**Figure 6:**
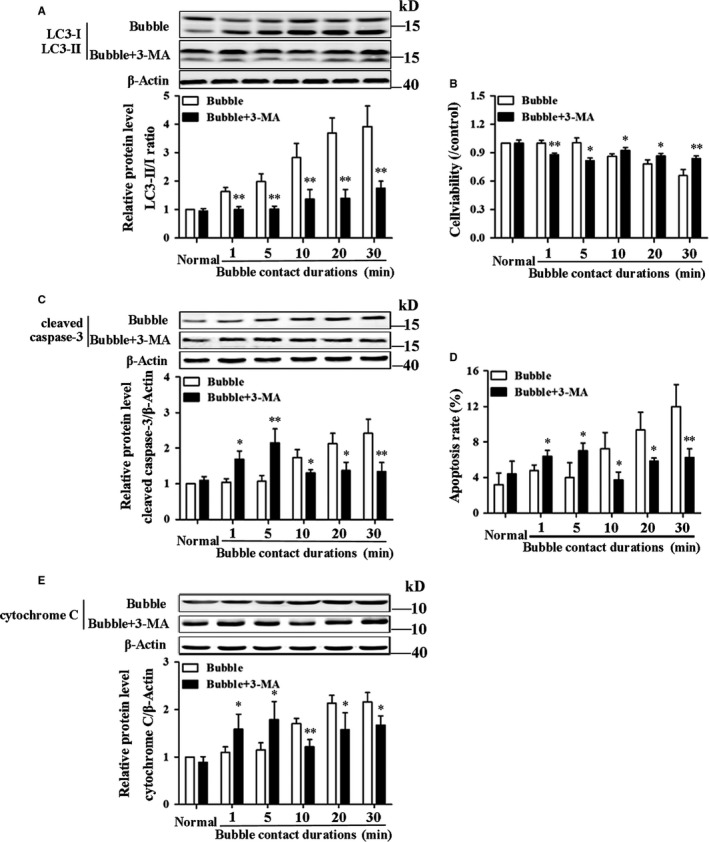
Effects of 3‐MA on bubble‐induced endothelial injury. HUVECs were pre‐treated with 3‐MA (5 mmol/L) for 24 h and exposed to bubbles for different durations. LC3‐II/I ratio (A) was determined at 8 h and cell viability (B), cleaved caspase‐3 (C), apoptosis rate (D) and cytochrome C (E) were determined at 12 h after bubble treatment. n = 4 for each group. ^*^ *P* < .05, ^**^ *P* < .01 vs the corresponding group without 3‐MA

With pre‐treatment of 3‐MA, the LC3‐II/I ratio significantly decreased in bubble treated cells (*P* < .01), and no difference was found between normal and 3‐MA pre‐treatment groups (Figure 6A, *P* > .05). As shown in Figure 6B‐E, cell viability was significantly increased, apoptosis rate, expressions of cytochrome C and cleaved caspase‐3 were remarkably decreased by 3‐MA pre‐treatment compared with the corresponding bubble alone groups of 10, 20 and 30 minutes (*P* < .05). 3‐MA induced a significant decrease in cell viability as well increases in apoptosis rate, cytochrome C and cleaved caspase‐3 in 1 or 5 minutes groups (*P* < .05).

### 3.3 Effects of a pre‐conditioning contact on a second long damaging contact by bubbles in HUVECs

**Figure 7:**
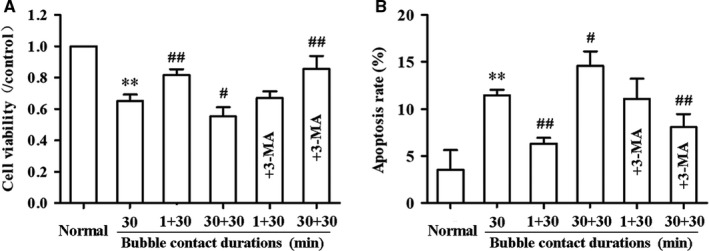
Effects of two consecutive bubble contact on endothelial injury. HUVECs were exposed to bubbles for 30 min following a pre‐conditioning for either 1 or 30 min with an 8 h interval. Cell viability (A) and apoptosis rate (B) were determined with or without 3‐MA (5 mmol/L) pre‐treatment for 24 h before the first bubble contact. Values are expressed as mean ± SD, n = 4 for each group. ^*^ *P* < .05, ^**^ *P* < .01 vs Normal. *^#^P* < .05, ^##^ *P* < .01 vs Bubble contact alone for 30 min

Based on the results above, the effects of two consecutive bubble contacts with an 8‐hours interval on HUVECs were observed. One and 30 minutes were selected as the pre‐conditioning protocols, with 30 minutes the damaging duration. Cell viability and apoptosis rate were determined to evaluate endothelial damage. The results are shown in Figure 7. Bubble pre‐conditioning for 1 minutes induced a significant increase in cell viability and decrease in apoptosis rate compared with no pre‐conditioning (*P* < .05), and 30 minutes of pre‐conditioning induced the opposite changes (*P* < .05). These changes were inhibited by 3‐MA (*P* < .05).

## 4 DISCUSSION

Autophagy is widely involved in physiological and pathological activities of endothelial cells.^11^ It is worth noting that it can be induced by mechanical and biochemical factors, including shear stress, calcium and oxidative stress,^15^, ^16^, ^17^ which are also influenced by decompression‐induced intravascular bubbles.^18^, ^19^, ^20^, ^21^, ^22^ We speculated that autophagy was probably involved in bubble‐induced endothelial injury. The autophagosome, featuring double‐membrane structures, detected by TEM, is a ‘gold standard’ of autophagy, and increases of LC3‐II/I ratio and Beclin‐1, and a decrease of P62, are widely accepted as biomarkers of enhanced autophagy. The former indicates the formation of autophagosome, while the latter reflects the degradation of autophagosome.^26^

In this study, the number of autophagosomes, LC3‐II/I ratio and Beclin‐1 significantly increased, and P62 decreased, after bubble treatment for different durations in HUVECs. LC3‐II/I ratio, Beclin‐1 and P62 reached their respective peaks/nadir at 8 hours and remained significantly changed at 24 hours after bubble treatment for 30 minutes. Linear regression revealed the peak levels of LC3‐II/I ratio and Beclin‐1 correlated positively, while the valley value of P62 correlated negatively, with bubble contact durations of 30 minutes or less. All these results suggested that autophagy could be induced by bubbles in a proportional linear relationship. There was a significant delay in the change for P62 compared with LC3‐II/I ratio and Beclin‐1, supporting that the degradation of autophagosome takes longer than its formation.^26^ Among the three indices, LC3‐II/I ratio had the strongest correlation, so it was used as an indicator of the degree of autophagy activation.

Bubble size may affect the correlation between autophagy activation and bubble contact duration. The diameter of bubbles was mainly influenced by the volume ratio of mixed gas to medium. In present study, the ratio we adopted was 0.5 and bubble formation showed low variation with diameters around 102 ± 16 μm.

Autophagy plays an important role in endothelial injury in various cardiovascular diseases.^27^ As a basal cellular process response to many stressors, autophagy may represent a cell protection mechanism to guarantee an efficient cytosolic clearance and maintain bioenergetics homoeostasis.^28^ However, under pathological conditions, impaired or over‐activated autophagy promotes cell injuries by accumulating intracellular damaged components, inducing excessive oxidative stress and promoting apoptosis death.^29^ In present study, there was a close relationship between LC3‐II/I ratio and endothelial injury, indicating that autophagy was involved in bubble‐induced endothelial dysfunction. The results of pre‐treatment with autophagy inhibitor 3‐MA showed that it inhibited the increase of LC3‐II/I ratio and significantly alleviated endothelial injury caused by microbubble contacts for 10, 20 and 30 minutes, confirming that autophagy promoted endothelial injury under these microbubble contact periods.

Surprisingly, 3‐MA pre‐treatment induced obvious endothelial damage following bubble contacts for 1 or 5 minutes, indicating that autophagy induced by 1 or 5 minutes of bubble contacts could exert beneficial effects on HUVECs against bubble stimuli. It has been reported that ischaemic treatment for 6 hours did not result in injuries of cortex tissues in rats, while inhibiting autophagy induced significant damage.^30^ The possible explanation is that autophagy contributes to the elimination of apoptotic‐competent mitochondria or other functionally redundant organelles and the maintenance of energy metabolism; thus, cells can counteract injuries under certain stimuli.^31^

To confirm the beneficial effects of autophagy induced by bubble contacts for short durations, we further observed the effects of two successive bubble contacts on endothelial function. The results showed that a pre‐conditioning bubble contact for 1 minutes significantly alleviated, while 30 minutes aggravated, endothelial injury caused by a second long damaging contact. These results confirmed that autophagy plays a biphasic role in bubble‐induced endothelial damage. Together with the relationship between autophagy and bubble contact duration, it further indicates that the biphasic effects of autophagy are related to the degree of autophagy activation, that is autophagy protein levels. In present study, the critical point for autophagy to play different roles might be between 1.95 ± 0.25‐fold (5 minutes group) and 2.53 ± 0.38‐fold (10 minutes group) of normal levels for LC3‐II/I ratio in HUVEC.

We also observed that endothelial damage caused by two consecutive 30 minutes bubble contact was reduced by 3‐MA pre‐treatment before the first contact when compared with the single bubble contact for 30 minutes. A possible reason for this may be that the LC3‐II/I ratio decreased to 1.75 ± 0.25‐fold of normal value, which was between the changes induced by bubble contacts for 1 and 5 minutes (1.63 ± 0.14‐fold and 1.98 ± 0.28‐fold, respectively). Thus, autophagy may protect endothelial cells against subsequent bubble damage.

Increased tolerance to DCS has been reported in regular divers, but the mechanism is not fully understood.^32^, ^33^ It has been found that a short ischaemic pre‐conditioning can protect endothelia against subsequent ischaemia‐reperfusion injuries by moderately up‐regulating autophagy.^34^ As the above results show, moderate up‐regulated autophagy by bubble contact for 1 minutes could protect HUVECs against damaging bubble stimuli. For regular divers, even if decompression protocols are strictly followed during each dive, bubbles may still form in the body,^35^ which may protect endothelial cells against future injuries through moderately up‐regulating autophagy. As endothelial targeting protection has been shown to prevent DCS,^5^, ^6^, ^7^ we speculate that increased tolerance to DCS in regular divers might be, at least in part, related to moderate up‐regulation of endothelial autophagy. Related rat‐model experiments are currently underway. For DCS patients, excessive autophagy of endothelia in vivo could also be a potential target of treatment.

In present study, bubble contact was performed at rest while in fact intravascular bubbles were flowing in one direction with blood in vivo. In addition to direct contact, changes in flow shear stress caused by bubbles may also be involved in endothelial injury.^18^, ^19^ Inflammatory responses caused by bubble‐induced activation of endothelial cells and leucocytes also aggravate endothelial injury in vivo.y^10^, ^18^ Thus, different modes of bubble action may lead to differences in the contact duration needed to induce obvious endothelial dysfunction and autophagy activation, both in vitro and in vivo.

In conclusion, autophagy was involved in bubble‐induced endothelial injury and played a biphasic role in the process. Furthermore, previous mild bubble stimulation alleviated subsequent endothelial injury caused by a second large intensity contact through moderately up‐regulating autophagy, which might be the mechanism of increased tolerance to DCS in divers who dive frequently. Finally, the biphasic role of autophagy makes it a potential target for preventing and treating DCS.

## CONFLICT OF INTEREST

All authors declare that they have no conflict of interest.

## AUTHORS CONTRIBUTIONS

WX, MW, KZ and SN designed the experiments. MW, KZ, SN and GH conducted the experiments. WX, MW, KZ, SN, GH, HY, CH and PB analysed the data and interpreted the results. WX, MW, KZ, GH and HY wrote the manuscript and prepared all of figures. CH and PB conceived the study and revised the manuscript. All authors read and approved the final version of the manuscript.

## Data Availability

The data in this paper are available from the corresponding author on reasonable requests.
